# Real-World Treatment Patterns and Outcomes Among Patients with Early Non-Small Cell Lung Cancer

**DOI:** 10.3390/curroncol32040239

**Published:** 2025-04-19

**Authors:** Jennifer D. Deem, Zsolt Hepp, Joshua J. Carlson

**Affiliations:** 1 CHOICE Institute, School of Pharmacy, University of Washington, Seattle, WA 98195, USA; carlsojj@uw.edu; 2 Pfizer Inc., New York, NY 10001, USA; zsolt.hepp@pfizer.com

**Keywords:** early NSCLC, treatment patterns, real-world, patient outcomes

## Abstract

Worldwide, about two million people are diagnosed with lung cancer each year, 85% of whom have non-small cell lung cancer (NSCLC). Recent progress in treating advanced/metastatic NSCLC with targeted therapies has shifted attention to early NSCLC (Stages I–IIIA) and perioperative (neoadjuvant and adjuvant) systemic therapies. However, our comprehension of how targeted therapeutics are incorporated into care and their impact on patient outcomes is just starting to unfold. Methods: This retrospective observational study used a US nationwide electronic health record-derived deidentified database spanning January 2019–March 2024 and aimed to describe (1) eNSCLC patient demographic and clinical characteristics, (2) real-world neoadjuvant and adjuvant use, and (3) patient outcomes. Results: The study population included 4841 Stage IB–IIIA NSCLC patients with a mean age of 70.9 ± 8.6 years. The majority (69.9%) received definitive treatment: surgery (*n* = 2280), definitive radiation (*n* = 320), or definitive chemoradiation (*n* = 783), while 30.1% (*n* = 1458) did not. Many definitive treatment patients received some perioperative systemic therapy (surgery: 52.6%, radiation: 52.2%, chemoradiation: 75.5%). Neoadjuvant use was limited in all groups (surgery: 8.2%, radiation: 6.1%, chemoradiation: 11.6%). Among the 54.6% receiving adjuvant, immune checkpoint inhibitors were the most common choice for definitive radiation (39.1%) and chemoradiation (73.7%) patients, while surgical patients predominantly received platinum-doublet therapy (37.0%). Surgical patient outcomes were similar across all groups, while definitive chemoradiation or radiation patients without systemic therapy had lower survival rates. Conclusions: In this study, we found that although the majority of patients underwent some form of definitive treatment, adjuvant use was limited, and neoadjuvant use was rarely included in care. A crucial initial step in improving patient outcomes is to understand and address the underutilization of neoadjuvant/adjuvant systemic therapy for eNSCLC patients.

## 1. Introduction

Lung cancer, the third most common cancer diagnosis and leading cause of cancer deaths in the US, is a significant health concern. About 85% of those diagnosed have non-small cell lung cancer (NSCLC), which is predominantly composed of either squamous or non-squamous (adenocarcinoma) subtypes, while the remaining fraction has large cell carcinoma. These histologically unrelated cancers have historically been treated similarly [1,2]. Over the last 15 years, targeted systemic treatments, including immunotherapy (IO), tyrosine kinase inhibitors (TKIs), and immune checkpoint inhibitors (ICIs), have improved survival from months to years in advanced NSCLC [2,3,4]. Due to their success in the later stages of NSCLC, the focus is now on earlier stages, with several clinical trials in the neoadjuvant and adjuvant space aiming to extend remission or achieve a cure.

Only 23% of NSCLC is caught “early”, prior to the development of metastatic disease (www.lung.org), when the course of treatment includes promising curative intent or definitive options [3]. The most common and effective definitive option is surgical resection, which results in conditional survival at five years of ~85% for patients with fully resectable Stage I tumors [5]. However, factors such as patient age (on average 65 years or older), poor health, and the technical limitations of surgical resection may classify some early tumors as inoperable [3]. Additionally, patients may forgo surgical resection despite clinical referral [6]. If surgery is not an option, definitive radiation or definitive chemoradiation, wherein a course of chemotherapy is provided either sequentially or concurrently with a definitive radiation course, remain important options [7].

**Figure 1:**
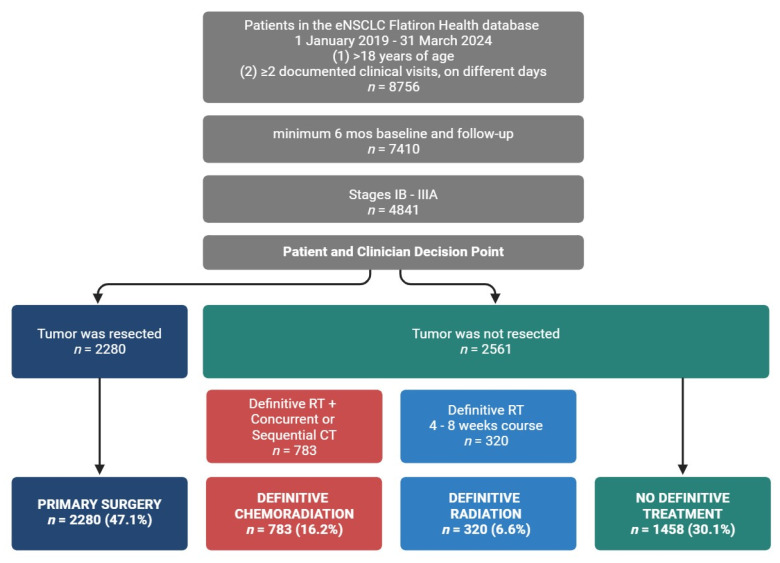
Curative intent treatment options define eNSCLC groups. Definitive treatment cohorts were as follows: primary surgery: patients who underwent primary surgical resection; definitive radiation: patients who received definitive radiation; definitive chemoradiation: patients who received definitive radiation with a concurrent or sequential course of chemotherapy; no definitive treatment: patients who did not receive definitive treatment.

On either side of these definitive options, the patient may be treated with systemic neoadjuvant and/or adjuvant therapies. However, up until 2020, the only approved systemic treatment for eNSCLC patients in the neoadjuvant/adjuvant systemic therapy setting was chemotherapy. Since then, the introduction of novel targeted systemic therapies based on cancer’s genetic signature (EGFR mutant, PDL1+, and/or ALK rearrangement) has underscored the role and importance of biomarker testing while rapidly transforming the landscape of eNSCLC care (Figure 1) [5]. Owing to the recent changes to care, improvements in real-world outcomes are not fully understood.

Recently published real-world treatment pattern studies in eNSCLC have been focused on a specific geographical area or country (e.g., Canada [8], Denmark [9], or Norway [10]), an associated genetic biomarker [11], or a specific clinical outcome (e.g., local recurrence [11] or post-resection use of adjuvant [12]). Many of these studies are also based on data prior to 2019 and do not reflect the numerous changes in eNSCLC care that have occurred since 2020 [3,13]. This has resulted in a fragmented and incomplete understanding of how eNSCLC patients are treated at the clinician level, given the challenges inherent in interpreting and applying these new therapeutics, particularly regarding timing and duration [1,7,14]. Given the rapidly changing landscape of neoadjuvant/adjuvant systemic therapy care options in the setting of eNSCLC, we used real-world data from US-based electronic health records to describe (1) the demographic and clinical characteristics of a real-world eNSCLC population (2) the treatment patterns of their care and (3) the impact of care on survival outcomes of patients diagnosed with eNSCLC. The results from this work can help inform clinical care and improve alignment between promising clinical trial evidence and real-world adoption of innovative treatments in eNSCLC.

## 2. Materials and Methods

### 2.1. Study Design and Data Source

This retrospective observational study used the nationwide, longitudinal Flatiron Health electronic health record (EHR)-derived deidentified database, comprising patient-level structured and unstructured data curated via technology-enabled abstraction [15,16]. During the study period, the deidentified data originated from approximately 280 US cancer clinics (~800 sites of care). The data are deidentified and subject to obligations to prevent reidentification and protect patient confidentiality. The database includes demographic and diagnostic information (e.g., stage, pathology, molecular information, and radiology), the extent of disease, lab values, treatments (e.g., line of therapy, dosing, and regimens), and patient outcomes. Dates of death are obtained from a composite mortality variable comprising the EHR structured data linked to mortality data, publicly available obituaries, and the Social Security Death Index and are resolved to the month of death [17].

### 2.2. Population

Of the 8756 patients with eNSCLC included in the real-world database and study time frame, a total of 4841 met inclusion criteria and were grouped according to curative intent or definitive treatment (Figure 1). Definitive treatment cohorts were patients classified as receiving primary surgical resection or non-surgical patients with receipt of a definitive course of radiation. We broadened the NCCN definition for definitive radiation (6–7 weeks) [7] to include patients who completed a radiation course lasting more than five but no more than nine weeks. Patients who received definitive radiation were stratified further according to receipt of a concurrent or sequential course of chemotherapy, with sequential chemotherapy and radiation occurring no more than seven weeks apart. Those who received both radiation and chemotherapy were classified as the definitive chemoradiation group. Those who did not receive chemotherapy were classified as the definitive radiation group. The remaining patients without definitive treatment were assigned to the no definitive treatment group. When classifying groups based on their use of perioperative systemic therapies, we relied on the oncologist-defined, rule-based system used by Flatiron to explicitly define both ‘neoadjuvant’ and ‘adjuvant’ lines of therapy. In general, treatment that occurs prior to definitive surgery or greater than 14 days prior to radiation therapy is considered neoadjuvant, while adjuvant is a treatment that occurs following surgery or radiation but prior to recurrence.

### 2.3. Population Inclusion/Exclusion Criteria

All patients in the study were at least 18 years old and diagnosed with lung cancer based on the following patient record criteria: (1) included ICD-9 162.x or ICD-10 C34x or C39.9 coding; (2) pathology record confirmed a diagnosis of early-stage (Stages IB–IIIA), non-metastatic disease, in line with the American Joint Committee on Cancer’s lung cancer staging system [18]; (3) had a minimum of two clinical visits on separate days; and (4) overall EHR data included a minimum of six months of baseline data and six months of follow-up data, indexed from their date of diagnosis. Of the 8756 patients with eNSCLC included in the real-world database from 1 January 2019 to 31 March 2024, a total of 4841 patients met the inclusion criteria.

### 2.4. Statistical Analysis

Statistical analyses were performed using R Language (version 4.4.0, R Project for Statistical Computing). Categorical variables were summarized by count and percent, and continuous variables as mean and standard deviation. Survival outcomes were analyzed using Kaplan–Meier analysis and median time to event, and 95% CIs were reported if achieved. Analyses were descriptive in nature, and no formal comparisons or hypotheses were tested.

### 2.5. Objective-Specific Analyses

#### 2.5.1. Baseline Demographic and Clinical Characteristics by Stage at Diagnosis and Definitive Treatment Group

We gathered baseline demographic and clinical characteristics using the date of diagnosis as the index date and the six months prior as the baseline period. If multiple instances of a characteristic were available, the instance closest to the diagnosis date was used. An exception was made for PDL1, EGFR, and ALK biomarker results, allowing specimens collected up to six months post-diagnosis, which is in line with recent literature [19]. Characteristics were stratified by stage at diagnosis and the presence or absence of definitive treatment, a course of treatment meant to eliminate or cure the cancer, as opposed to managing symptoms, e.g., complete resection of the primary tumor. Demographic characteristics included sex, age, US geographic region, treatment center and insurance type, and race/ethnicity. Clinical characteristics included baseline ECOG, smoking history, histological subtype (squamous or non-squamous), and three key biomarkers (ALK, EGFR, and PDL1).

#### 2.5.2. Use of Neoadjuvant/Adjuvant Systemic Therapies by Definitive Treatment Group and Stage at Diagnosis

Adjuvant treatment was defined as the initial treatment occurring after definitive treatment but before recurrence, while neoadjuvant treatments occurred after diagnosis but before definitive treatment. Treatment patterns were grouped by stage at diagnosis and the presence or absence of definitive treatment. Sankey plots, a type of flow diagram that visualizes the quantitative movement between “nodes”, were used to describe treatment patterns with neoadjuvant, definitive treatment, and adjuvant as the three “nodes” across which patients temporally progressed.

#### 2.5.3. Event-Driven Outcomes by Treatment Group and Neoadjuvant/Adjuvant Use

We defined a new index for this objective as the initiation of definitive treatment: primary surgery, definitive radiation, or definitive chemoradiation, allowing for the index date to be preceded by neoadjuvant treatment for some patients. Survival outcomes in line with those commonly included in oncology clinical trial design were estimated from the first day of definitive treatment, i.e., the date of resection, the first day of definitive radiation, or the first day of chemotherapy in the definitive chemoradiation course. Outcomes were organized by definitive treatment group and adjuvant and/or neoadjuvant treatments. Real-world overall survival was estimated from the initiation of definitive treatment to death from any cause, loss to follow-up, or study end. Real-world event-free survival was estimated from the initiation of definitive treatment until death from any cause, local or distant recurrence, loss to follow-up, or study end. For all survival plots, individuals were censored at the date of the last visit or study end, 31 March 2024, whichever occurred first. Survival analyses were performed using lognormal parametric distributions to model the survival times. To account for changes in approved targeted therapeutic options over the study period, definitive treatment cohorts were also segregated by year of diagnosis (rolling admissions), and subgroup analyses were performed accordingly.

## 3. Results

### 3.1. Baseline Demographic and Clinical Characteristics by Stage at Diagnosis and Definitive Treatment Group

To characterize the eNSCLC study population, patient demographics and clinical characteristics were grouped by definitive treatment (Table 1 and Table 2). The study included 4841 patients with an average age of 70.9 ± 8.6 years. Most patients were diagnosed at Stage IIIA (*n* = 1972, 40.7%), with the rest in Stage IB (*n* = 1529, 31.6%) and Stage II (*n* = 1340, 27.7%). The sample was 51.0% female, with patients diagnosed at earlier stages predominantly female (Stage IB, 54.7% female), while later stages had more male patients (Stage IIIA, 52.3% male). About 70% of the population was aged > 65, and ~90% had a history of smoking, with lower rates of smoking in patients diagnosed at early stages. Patients were divided into cohorts based on definitive treatment: primary surgery, definitive radiation, and definitive chemoradiation (Figure 1), according to NCCN guidelines [7].

Patients who received surgical resection were classified under the ‘Primary Surgery’ cohort (47.1%, *n* = 2280). Unlike other groups, surgical patients were predominantly female (53.6%) and Stage IB (43.7%). In line with patient health requirements for surgical resection and recovery, patients in the primary surgery group were less likely to have a history of smoking (past or current) (86.5%) compared to those receiving definitive radiation (95.0%), definitive chemoradiation (96.0%), or no definitive treatment (93.3%). Overall, patients were primarily diagnosed with non-squamous histology (61.6%), and a higher fraction of non-squamous patients underwent primary surgery (73.4%) compared to those receiving definitive radiation (51.6%), definitive chemoradiation (46.7%), or no definitive treatment (53.2%). A larger fraction of primary surgery patients had EGFR-positive status (13.7%) when compared to definitive radiation (3.4%), definitive chemoradiation (3.1%), and no definitive treatment patients (7.3%). For all groups, as the stage at diagnosis increased, biomarker testing rates increased from 45.5–51.3% to 66.4–72.8%.

Those who underwent a definitive course of radiation were categorized into the ‘Definitive Radiation’ cohort (6.6%, *n* = 320). The proportion of black patients was greater in this group (13.1% vs. 7.2–8.8% in other groups), and they were slightly older than surgical patients. Most patients receiving definitive radiation were diagnosed at Stage IIIA (80.6%). This group had the highest EGFR testing rates (63.1%) but low EGFR positivity (3.4%).

A separate cohort, termed ‘Definitive Chemoradiation’, was established for patients who, in addition to undergoing a definitive course of radiation therapy, were also administered chemotherapy either concurrently or in a sequential manner (16.2%, *n* = 783). This group had the highest fraction of patients with a smoking history (96.0%) and nearly a 1:1 male-to-female ratio. Most patients were diagnosed at Stage IIIA (78.3%), with less than 2% diagnosed at Stage IB. Like the definitive radiation group, they had high EGFR testing rates (62.8%) but low EGFR positivity (3.1%).

### 3.2. Use of Neoadjuvant/Adjuvant Systemic Therapies by Definitive Treatment Group and Stage at Diagnosis

We observed an increase in the administration of systemic adjuvant or neoadjuvant therapeutics (Table 3) corresponding to the severity of staging at diagnosis across all definitive treatment groups. The use of neoadjuvant therapy remained limited across all groups, irrespective of disease stage, definitive treatment modality, or combination with adjuvant therapy (surgery, 8.2%; definitive radiation, 11.6%; definitive chemoradiation, 6.1%). The absence of adjuvant/neoadjuvant therapy was more prevalent among patients undergoing surgical resection and definitive radiation therapy (47.4% and 47.8%, respectively), with 72.8% of Stage IB surgical patients receiving surgery alone. In contrast, only 24.5% of patients receiving definitive chemoradiation did not receive any neoadjuvant/adjuvant therapy.

Upon examining the subset of patients who received neoadjuvant/adjuvant systemic therapy (Table 4), we observed a greater diversity in treatment modalities in the neoadjuvant context compared to the adjuvant setting. This trend was consistent across all cohorts, including those who received neoadjuvant systemic therapy but did not proceed to definitive treatment. For patients treated with systemic neoadjuvant therapy, the most frequently administered neoadjuvants across all cohorts were either platinum doublet, such as cisplatin and pemetrexed (primary surgery, 20.9%; definitive radiation, 48.6%; definitive chemoradiation, 58.3%; no definitive treatment, 37.9%), or platinum doublet combined with an immune checkpoint inhibitor (ICI) (primary surgery, 43.3%; definitive radiation, 35.1%; definitive chemoradiation, 16.7%; no definitive treatment, 13.7%). Notably, a substantial proportion (10.7%) of surgical patients who received neoadjuvant therapy were administered a clinical study drug in this setting.

**Figure 2:**
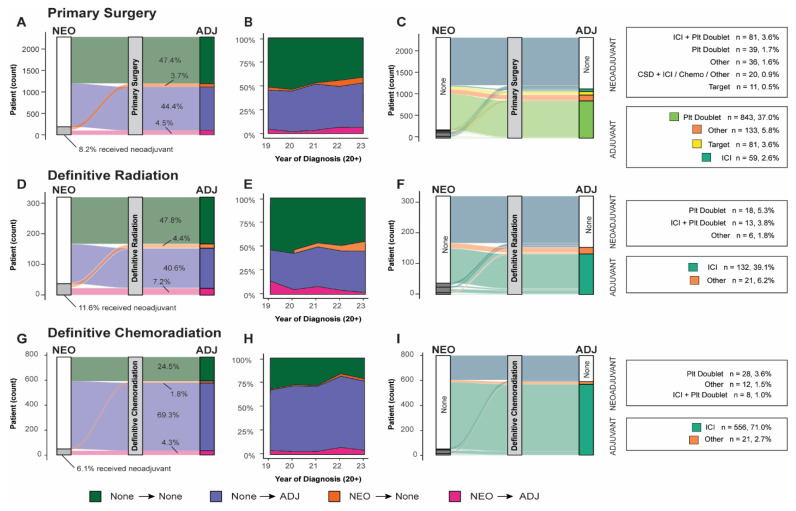
Neoadjuvant/adjuvant systemic therapy use by patients by definitive treatment: (**A**) Sankey diagram of all surgical patients organized by use of neoadjuvant or adjuvant. (**B**) Use of neoadjuvant, adjuvant, both, or none across all surgical patients organized by year of diagnosis. (**C**) Sankey diagram of specific systemic neoadjuvant and adjuvant lines of treatment reaching > 5% use threshold. (**D**) Sankey diagram of all definitive chemoradiation patients organized by use of neoadjuvant or adjuvant. (**E**) Use of neoadjuvant, adjuvant, both, or none across definitive chemoradiation treatment group organized by year of diagnosis. (**F**) Sankey diagram of specific systemic neoadjuvant and adjuvant lines of treatment reaching > 5% use threshold. (**G**) Sankey diagram of all definitive radiation patients organized by use of neoadjuvant or adjuvant. (**H**) Use of neoadjuvant, adjuvant, both, or none across definitive radiation treatment groups organized by year of diagnosis. (**I**) Sankey diagram of specific systemic neoadjuvant and adjuvant lines of treatment reaching > 5% use threshold. (ICI: immune checkpoint inhibitor; Plt: platinum-based chemotherapy; ADJ: adjuvant; NEO: neoadjuvant.

A significant fraction of patients who received neoadjuvant therapy subsequently received adjuvant therapy across all definitive treatment groups. Of the 8.2% (*n* = 187) of neoadjuvant-receiving surgery patients, 55.1% went on to receive adjuvant therapy. Similarly, 62.2% of neoadjuvant-receiving definitive radiation patients and 70.8% of neoadjuvant-receiving definitive chemoradiation patients proceeded to adjuvant therapy. The use of systemic adjuvant therapy was higher in more recently diagnosed patients (Figure 2B,E,H).

For patients who received adjuvant therapy, ICI monotherapy emerged as the predominant therapeutic approach for those undergoing definitive radiation or chemoradiation, with 86.3% and 96.4%, respectively, receiving this therapy. Conversely, only 5.3% of surgical patients were administered ICI monotherapy in this context. Surgical patients were more commonly administered platinum doublet, and 7.3% received targeted therapy (Table 4). The administration of targeted systemic therapeutics was higher for surgical patients (8.0%) and in the neoadjuvant context for patients who did not receive definitive treatment (11.2%).

### 3.3. Event-Driven Outcomes by Treatment Group and Neoadjuvant/Adjuvant Use

**Figure 3:**
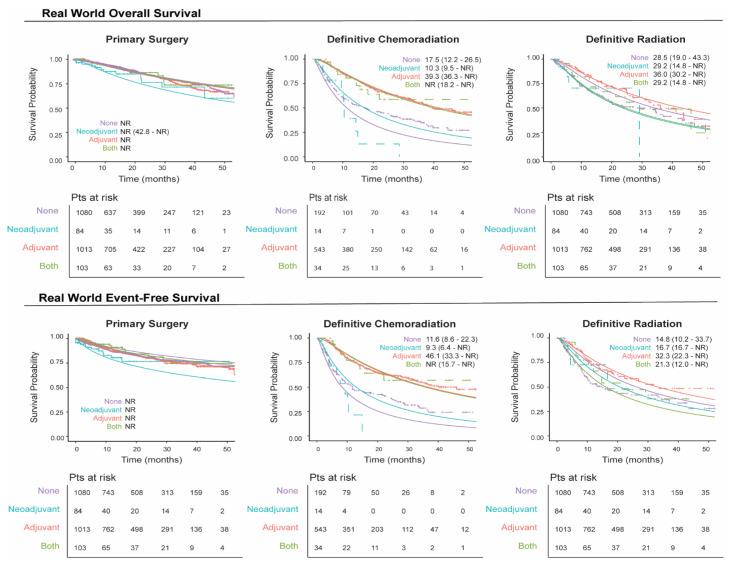
Real-world overall and event-free survival by definitive treatment groups stratified by neoadjuvant/adjuvant systemic therapy. The solid line represents the fitted survival plot, an estimate of the survival function. The dashed lines represent the observed survival data.

We plotted unmatched survival outcomes by definitive treatment group according to the receipt of neoadjuvant and/or adjuvant systemic therapy. In patients with resectable eNSCLC, those who received neoadjuvant and/or adjuvant therapy in conjunction with primary surgery generally exhibited a similar overall survival rate over time compared to those who did not receive such treatment (Figure 3), which may be due to bias by indication, with patients diagnosed at a later stage more likely to receive perioperative therapy. Conversely, in patients treated with definitive radiation or chemoradiation, survival outcomes were higher for those who received adjuvant systemic therapy relative to those who did not receive neoadjuvant and/or adjuvant systemic therapy (Figure 3). These patterns were also reflected in the event-free survival (EFS) rates (Figure 3), where patients who underwent tumor resection with neoadjuvant and/or adjuvant systemic therapy experienced a similar EFS over time, while those who received adjuvant therapy following definitive radiation or chemoradiation demonstrated higher overall EFS over time.

The 1-year survival rate for patients who underwent surgical resection without neoadjuvant and/or adjuvant systemic therapy, regardless of the stage at diagnosis, remained stable and ranged from 94.7% to 97.9% (Supplemental Figure S1 and Tables S4 and S5). For patients undergoing definitive chemoradiation without neoadjuvant and/or adjuvant systemic therapy, 1-year survival rates were generally lower, ranging from 37.9% to 71.7%. However, patients in this group who received adjuvant therapy exhibited higher 1-year survival rates, ranging from 80.0% to 100%. Similar trends were observed in 2-year survival rates. A comparable pattern was seen in patients who received definitive radiation alone, although their survival rates were slightly higher overall, ranging from 66.3% to 96.8%.

## 4. Discussion

Using deidentified data from US electronic health records, we identified a cohort of 4841 real-world eNSCLC patients in our study. We grouped patients by curative intent or definitive treatment (primary surgery, definitive radiation, and definitive chemoradiation) as defined by NCCN guidelines [7] and evaluated the use of systemic neoadjuvant and adjuvant therapies by stage at diagnosis. The NCCN guidelines for early-stage NSCLC recommend surgical resection for healthy patients to completely remove the tumor. For those unable to undergo surgery, definitive radiation is advised to control the tumor locally or if the operability of the tumor is limited. Definitive chemoradiation is used for more aggressive tumors or when combined treatment is needed for better outcomes. These decisions are based on patient health, tumor characteristics, and treatment goals. We found that the majority of patients underwent surgical resection of the primary tumor, while distinctly smaller fractions underwent courses of definitive radiation with or without chemotherapy. Additionally, over a quarter of the population received treatments that did not meet the criteria for definitive therapy. A significant fraction did not receive systemic perioperative therapies, especially surgery (*n* = 1080, 47.4%) and definitive radiation (*n* = 153, 47.8%) patients, and to a lesser extent, chemoradiation patients (*n* = 192, 24.5%). Targeted treatments were prevalent in patients undergoing definitive radiation or chemoradiation, with adjuvant ICI monotherapy being the main systemic treatment. Conversely, fewer surgical patients received neoadjuvant therapy, and platinum-based chemotherapy was the primary adjuvant treatment, with only a few receiving ICIs. This lower ICI uptake in surgical patients may be due to lower biomarker testing for early diagnoses, generally better patient health, physician preference, patient access, or the perception of immunotherapies as new and unproven.

Survival rates were steady for surgery patients, regardless of neoadjuvant/adjuvant systemic therapy, while definitive chemoradiation and radiation patients showed higher survival with adjuvant therapy. Despite these qualitative differences between outcomes, we are unable to draw any conclusions without performing correctly matched and bias-adjusted comparison studies. When considering how best to design these analyses, it is important to note NCCN guidance on both definitive treatment choice and neoadjuvant/adjuvant use, as treatment decision-making is highly dependent on underlying patient health, resectability of the tumor, and stage at diagnosis. As an example, neoadjuvant use is indicated only for patients Stage II or later. The surgery population included a sizeable Stage IB fraction (43.7%), suggesting that our neoadjuvant-treated surgery group may be more heavily weighted with patients diagnosed at later stages such that it obscures any benefit provided by neoadjuvant use.

Historically, clinical trials for eNSCLC focused on resectable candidates. In the neoadjuvant setting, standard care for Stage II+ patients has been platinum-based chemotherapy. However, neoadjuvant options for resectable patients now include nivolumab and durvalumab following chemotherapy, regardless of PDL1 status, approved in 2022 and 2024, respectively, or pembrolizumab, approved in 2022, for those without contraindications for ICI use. In the adjuvant setting, in 2023, the FDA approved pembrolizumab as an adjuvant for up to one year. Additional recent approvals in the adjuvant setting include nivolumab, approved in 2024; durvalumab, approved in 2018; atezolizumab, approved in 2021; and pembrolizumab, approved in 2023, for resectable Stage II+ patients. Moreover, osimertinib, approved in 2020, and alectinib, approved in 2024, have been approved for resectable patients positive for EGFR [14] and ALK mutations, respectively. Monitoring the progression of how these new therapies impact the overall use of neoadjuvant and adjuvant therapeutics and the subsequent effect on real-world patient outcomes is imperative.

While primarily descriptive, our results underscore the unaddressed needs of patients diagnosed with eNSCLC. Perioperative treatments can enhance surgical outcomes, improve survival rates, and contribute to personalized cancer care. However, their use requires understanding the patient’s condition, tumor characteristics, and treatment risks and benefits [1,3,19]. Despite increased biomarker testing, the use of ICI and targeted therapies remains below those in other advanced diseases [20], highlighting the need to address access to testing. While guidelines for eNSCLC care are available, actual patient care varies based on disease severity, physician and care team decision-making, patient preferences [6], overall health, and access to care [1]. Our results underscore the unaddressed needs of eNSCLC patients and the opportunity for new perioperative therapies to have significant impacts in the future [1,3]. Efforts to increase access to perioperative therapies could improve the quality of life and overall outcomes for patients during this “curable” phase of the disease.

## 5. Limitations

Given the continuous emergence of newly approved targeted therapies, our analyses are in the nascent stage. A more comprehensive interpretation might be achieved by examining data in subgroups delineated by the year of diagnosis. Our classification of the three definitive treatment groups is based on curative intent, but this may not perfectly align with the therapeutic goals of the medical team. Our framework is restricted by inclusion criteria, only including patients who completed their prescribed course of radiation or chemotherapy. Patients who discontinued treatment prematurely were included in the ‘No Definitive Treatment’ group. A detailed investigation of treatment options for patients without curative intent would provide valuable insights.

Although our dataset spans from January 2019 to March 2024, the TNM classification system underwent an impactful update in 2016, when the clinical trials on which many of the discussed key drugs were running. Importantly, some tumors that might have been classified as Stage IB based on tumor size were reclassified as Stage II in the revised 8th edition. Specifically, based on the 7th edition, nivolumab and pembrolizumab were indicated for resectable Stage IB patients, while under the 8th edition, they are only approved for resectable Stage II+ patients. We do not know how this shift may have impacted physician decision-making or how it might be reflected in our results, particularly in Stage IB patients.

The racial and ethnic composition of patients with eNSCLC in the real-world database aligns closely with national averages for the White/Caucasian population (68.7%) but underrepresents the Black (8.1% vs. 12.5%) and Asian (2.1% vs. 5.8%) populations in the US. Over 40% of patients were from the southern US, potentially indicating a limitation due to the overrepresentation of oncology clinics in this region. However, previous research suggests that this database is generally representative of the overall US population distribution [16]. In addition, our study provides an incomplete picture of underlying patient health. We are limited to smoking history, age, and ECOG values at baseline for approximately 75% of patients overall and only 50% of Stage IB patients. Additionally, the calculation of the Charlson Comorbidity Index, which would add context to our findings, is hampered by the fact that we only have patient data from oncology clinics and academic centers and may not represent a patient’s full medical history.

Our objective was to describe differences in the use of neoadjuvant and adjuvant systemic therapeutics within definitive treatment groups. Aligning patient care from the initiation of any treatment presented a challenge in assessing event-driven outcomes (1-year, 2-year, overall survival, and event-free survival). We defined our index as the initiation of definitive treatment: primary surgery, definitive radiation, or definitive chemoradiation, allowing that for some patients, the index date could be preceded by neoadjuvant treatment.

Our study continuously enrolled eNSCLC patients who typically have a lifespan exceeding our study duration, resulting in preliminary results that will become more robust over time. The potential for direct comparison or interpretation of our survival outcomes is constrained by non-random censoring due to differential follow-up times. Future periodic re-analyses and collaborative studies pooling data from multiple sources will strengthen our ability to accurately interpret and statistically analyze the data to improve the reliability of our results.

## 6. Conclusions

In this retrospective observational study of NSCLC diagnosed at stages I-IIIA, 47.1% of the patients underwent surgical resection, while ~22% completed a course of definitive radiation with (6.6%) or without chemotherapy (16.2%). The remaining patients underwent piecemeal courses of radiation or systemic treatment. On either side of these definitive options, we found that the use of systemic neoadjuvant was rare, and adjuvant use was limited despite growing evidence of its effectiveness for eNSCLC patients [1,3]. It is noteworthy that throughout the study period, the approved perioperative systemic therapies for eNSCLC patients evolved from solely chemotherapy to include six targeted systemic therapeutics. These advancements were predominantly for biomarker-positive resectable eNSCLC and were indicated for specific stages and definitive treatments. Future research, aligned with the approval of new therapies, may elucidate how to effectively integrate neoadjuvant/adjuvant therapies into eNSCLC patient care by identifying discrepancies between clinical practice and guideline changes [1,14]. Indeed, the potential for recurrence and progression to metastatic disease remains significant for eNSCLC patients, even after definitive treatment with or without adjuvant/neoadjuvant therapy [19,21]. This study underscores the unmet needs of this population, highlighting the necessity for better application of currently approved therapies in clinical practice, as well as the continued development of novel therapies.

## Figures and Tables

**Table 1:** Baseline patient demographics and clinical characteristics by stage at diagnosis. Definitive treatment groups defined by color: Primary Surgery, light blue; Definitive Radiation, teal; Definitive Chemoradiation, red; No Definitive Treatment, green.

Stage at Diagnosis		Any Stage	Stage IB	Stage II	Stage IIIA
**Total**		4841	1529	1340	1972
**Patient Demographics**					
Definitive Treatment (*n*, %)	Primary Surgery	2280 (47.1)	997 (65.2)	775 (57.8)	508 (25.8)
	Definitive Radiation	320 (6.6)	12 (0.8)	50 (3.7)	258 (13.1)
	Definitive Chemoradiation	783 (16.2)	13 (0.9)	157 (11.7)	613 (31.1)
	No Definitive Treatment	1458 (30.1)	507 (33.1)	358 (26.7)	593 (30.1)
Gender (*n*, %)	Male	2374 (49.0)	692 (45.3)	650 (48.5)	1032 (52.3)
	Female	2467 (51.0)	837 (54.7)	690 (51.5)	940 (47.7)
Age at diagnosis (*n*, %)	19–49	68 (1.5)	14 (0.9)	16 (1.2)	32 (1.6)
	50–64	1038 (21.4)	294 (19.2)	286 (21.3)	458 (23.2)
	65–74	1734 (35.8)	560 (36.6)	466 (34.8)	708 (35.9)
	74–84	1909 (39.4)	632 (41.3)	542 (40.4)	735 (37.3)
	85+	98 (2.0)	29 (1.9)	30 (2.2)	39 (2.0)
	Mean (SD)	70.9 (8.6)	71.4 (8.2)	71.1 (8.7)	70.4 (8.8)
Race/Ethnicity (*n*, %)	White/Caucasian	3284 (67.8)	1045 (68.3)	923 (68.9)	1316 (66.7)
	Black/African American	390 (8.1)	109 (7.1)	109 (8.1)	172 (8.7)
	Asian	102 (2.1)	28 (2.1)	28 (2.1)	35 (1.8)
	Other Race	296 (6.1)	79 (5.2)	78 (5.8)	139 (7.0)
	Not documented	768 (15.9)	257 (16.8)	202 (15.1)	309 (15.7)
Geographic region (*n*, %)	Midwest	512 (10.6)	144 (9.4)	145 (10.8)	223 (11.3)
	South	2063 (42.6)	608 (39.8)	560 (41.8)	895 (45.4)
	Northeast	496 (10.2)	154 (10.1)	154 (11.5)	188 (9.5)
	West	623 (12.9)	204 (13.3)	185 (13.8)	234 (11.9)
	Other Territories	26 (0.5)	11 (0.7)	6 (0.4)	9 (0.5)
	Not documented	1121 (23.2)	408 (26.7)	290 (21.6)	423 (21.5)
Treatment Center (*n*, %)	Academic	1042 (21.5)	384 (25.1)	270 (20.1)	388 (19.7)
	Community	3799 (78.5)	1145 (74.9)	1070 (79.9)	1584 (80.3))
Insurance Type (*n*, %)	Commercial	3588 (74.1)	1126 (73.6)	996 (74.3)	1466 (74.3)
	Medicaid	131 (2.7)	31 (2.0)	43 (3.2)	57 (2.9)
	Medicare	706 (14.6)	261 (17.1)	186 (13.9)	259 (13.1)
	Other Gov’t Program	161 (3.3)	51 (3.3)	44 (3.3)	66 (3.3)
	Unknown	193 (4.0)	50 (3.3)	54 (4.0)	89 (4.5)
	Patient Assistance Program	62 (1.3)	10 (0.7)	17 (1.3)	35 (1.8)
**Clinical Characteristics**					
ECOG (*n*, %)	0	1575 (32.5)	514 (24.6)	433 (32.3)	628 (31.8)
	1	1572 (32.5)	408 (19.5)	473 (35.3)	691 (35.0)
	2	411 (8.5)	101 (4.8)	115 (8.6)	195 (9.9)
	3+	109 (2.3)	21 (1.0)	35 (2.6)	53 (2.7)
	Not documented	1174 (24.3)	1044 (50.0)	284 (21.2)	405 (20.5)
Smoking History (*n*, %)	History of smoking	4389 (90.7)	1322 (86.5)	1231 (91.9)	1836 (93.1)
	No history of smoking	450 (9.3)	207 (13.5)	108 (8.1)	135 (6.8)
	Not documented	2 (0.04)	--	1 (0.07)	1 (0.1)
**Tumor Characteristics**					
Histological Subtype (*n*, %)	Non-Squamous	2980 (61.6)	1093 (71.5)	798 (59.6)	1089 (55.2)
	Squamous	1739 (35.9)	417 (27.3)	513 (38.3)	809 (41.0)
	Unknown/Not specified	122 (2.5)	19 (1.2)	29 (2.2)	74 (3.8)
PDL1 Staining (*n*, % of tested)	Patients Tested (*n*, % total)	2861 (59.1)	714 (46.7)	837 (62.5)	1310 (66.4)
	<1% staining	642 (22.4)	167 (23.4)	174 (20.8)	288 (22.0)
	1–49% staining	957 (33.4)	250 (35.0)	305 (36.4)	421 (32.1)
	≥50% staining	598 (20.9)	113 (15.8)	185 (22.1)	301 (23.0)
	Inconclusive	664 (23.2)	184 (25.8)	173 (20.7)	300 (22.9)
EGFR Status (*n*, % of tested)	Patients Tested (*n*, % total)	3147 (75.9)	785 (51.3)	927 (69.2)	1435 (72.8)
	Positive	306 (9.7)	111 (14.1)	83 (9.0)	112 (7.8)
	Negative	2607 (82.8)	600 (76.4)	774 (83.5)	1233 (85.9)
	Inconclusive/Unknown	234 (7.4)	74 (9.5)	70 (7.6)	90 (6.3)
ALK Status (*n*, % of tested)	Patients Tested (*n*, % total)	2992 (61.8)	695 (45.5)	864 (64.5)	1433 (72.7)
	Positive	47 (1.6)	7 (1.0)	20 (2.3)	20 (1.4)
	Negative	2710 (90.6)	615 (88.5)	781 (90.4)	1314 (91.7)
	Inconclusive/Unknown	235 (7.9)	73 (10.5)	63 (7.3)	99 (6.9)

**Table 2:** Baseline patient demographics and clinical characteristics by definitive treatment group. Definitive treatment groups defined by color: Primary Surgery, light blue; Definitive Radiation, teal; Definitive Chemoradiation, red; No Definitive Treatment, green.

Definitive Treatment Group		Primary Surgery (n = 2280)	Definitive Radiation (n = 320)	Definitive Chemoradiation (n = 783)	No Definitive Treatment (n = 1458)
**Patient Demographics**					
Gender (*n*, %)	Male	1058 (46.4)	168 (52.5)	397 (50.7)	751 (51.5)
	Female	1222 (53.6)	152 (47.5)	386 (49.3)	707 (48.5)
Age at diagnosis (*n*, %)	19–49	39 (2.1)	3 (0.9)	6 (0.8)	14 (1.0)
	50–64	600 (26.3)	75 (23.4)	164 (20.9)	199 (13.6)
	65–74	918 (40.3)	106 (33.1)	277 (35.4)	433 (29.7)
	74–84	712 (31.2)	131 (40.9)	333 (42.5)	733 (50.3)
	85+	11 (0.5)	5 (1.6)	<5 (0.4)	79 (5.4)
	Mean (SD)	69.1 (8.3)	70.8 (8.4)	71.0 (8.3)	73.7 (8.6)
Race/Ethnicity (*n*, %)	White	1596 (70.0)	200 (62.5)	537 (68.6)	951 (65.2)
	Black/African American	163 (7.2)	42 (13.1)	69 (8.8)	116 (8.0)
	Asian	62 (2.7)	7 (2.2)	8 (1.0)	25 (1.7)
	Other	117 (5.1)	13 (3.8)	55 (7.0)	112 (7.7)
	Not documented	342 (15.0)	58 (18.1)	114 (14.6)	254 (17.4)
Geographic region (*n*, %)	Midwest	243 (10.7)	30 (9.4)	99 (12.6)	140 (9.6)
	South	845 (37.1)	157 (49.1)	414 (52.9)	647 (44.4)
	Northeast	245 (10.7)	37 (11.6)	67 (8.6)	147 (10.1)
	West	273 (12.0)	33 (10.3)	85 (10.9)	232 (15.9)
	Other Territories	12 (0.5)	--	4 (0.5)	10 (0.7)
	Not documented	662 (29.0)	63 (19.7)	114 (14.6)	282 (19.3)
Treatment Center (*n*, %)	Academic	625 (27.4)	56 (17.5)	103 (13.2)	258 (17.7)
	Community	1655 (72.6)	264 (82.5)	680 (86.8)	1200 (82.3)
Insurance Type (*n*, %)	Commercial	1710 (75.0)	215 (67.2)	589 (75.2)	1074 (73.7)
	Medicaid	44 (1.9)	10 (3.1)	31 (4.0)	46 (3.2)
	Medicare	349 (15.3)	47 (14.7)	100 (12.8)	210 (14.4)
	Other Gov’t Program	50 (2.2)	22 (6.9)	29 (3.7)	60 (4.1)
	Unknown	97 (4.3)	18 (5.6)	26 (3.3)	51 (3.7)
	Patient Assistance Program	31 (1.4)	8 (2.5)	8 (1.0)	15 (1.0)
**Clinical Characteristics**					
Stage at Diagnosis (*n*, %)	Stage IB	997 (43.7)	12 (3.8)	13 (1.7)	507 (34.8)
	Stage II	775 (34.0)	50 (15.6)	157 (20.0)	358 (24.6)
	Stage IIIA	508 (22.3)	258 (80.6)	613 (78.3)	593 (40.7)
ECOG (*n*, %)	0	829 (36.4)	88 (27.5)	288 (37.3)	370 (26.7)
	1	651 (28.6)	108 (33.8)	325 (42.1)	488 (35.2)
	2	100 (4.4)	23 (7.2)	89 (11.5)	199 (14.4)
	3+	17 (0.1)	9 (3.9)	17 (2.2)	66 (4.8)
	Not documented	683 (30.0)	92 (28.8)	53 (6.9)	263 (19.0)
Smoking History (*n*, %)	History of smoking	1973 (86.5)	304 (95.0)	752 (96.0)	1360 (93.3)
	No history of smoking	306 (13.4)	16 (5.0)	31 (4.0)	97 (6.7)
	Not documented	1 (0.04)	--	--	1 (0.1)
**Tumor Characteristics**					
Histological Subtype (*n*, %)	Non-Squamous	1674 (73.4)	165 (51.6)	366 (46.7)	775 (53.2)
	Squamous	584 (25.6)	139 (43.4)	379 (48.4)	637 (43.7)
	Unknown/Not specified	22 (1.0)	16 (5.0)	38 (4.9)	46 (3.2)
PDL1 Staining (*n*, %)	Patients Tested (*n*, % total)	1420 (62.3)	184 (57.5)	469 (60.8)	788 (57.0)
	<1% staining	317 (22.3)	39 (21.2)	104 (22.2)	182 (23.1)
	1–49% staining	513 (36.1)	56 (30.4)	139 (29.6)	249 (31.6)
	≥50% staining	275 (19.4)	41 (22.3)	125 (26.7)	157 (19.9)
	Inconclusive	315 (22.2)	48 (26.1)	101 (21.5)	200 (25.4)
EGFR Status (*n*, %)	Patients Tested (*n*, % total)	1615 (41.2)	202 (63.1)	485 (62.8)	845 (61.0)
	Positive	222 (13.7)	7 (3.4)	15 (3.1)	62 (7.3)
	Negative	1300 (80.5)	173 (85.6)	441 (90.9)	693 (82.0)
	Inconclusive/Unknown	93 (5.8)	22 (10.9)	29 (6.0)	90 (10.7)
ALK Status (*n*, %)	Patients Tested (*n*, % total)	1461 (64.1)	197 (61.6)	493 (63.9)	841 (60.7)
	Positive	27 (1.8)	3 (1.5)	6 (1.2)	11 (1.3)
	Negative	1345 (92.1)	177 (89.8)	452 (91.7)	736 (87.5)
	Inconclusive/Unknown	89 (6.1)	17 (8.6)	35 (7.1)	94 (11.2)

**Table 3:** Neoadjuvant/adjuvant systemic therapy use by definitive treatment group and stage at diagnosis. Definitive treatment groups defined by color: Primary Surgery, light blue; Definitive Radiation, teal; Definitive Chemoradiation, red; No Definitive Treatment, green.

Stage at Diagnosis	Overall	Stage IB	Stage II	Stage IIIA
	4841	1529	1340	1972
**Primary Surgery, *n* = 2280**				
Surgery alone	1080 (47.4)	725 (72.8)	241 (31.1)	114 (22.4)
Neoadjuvant	84 (3.7)	37 (3.7)	24 (3.1)	23 (4.5)
Adjuvant	1013 (44.4)	193 (19.4)	482 (62.2)	338 (66.5)
Neoadjuvant + adjuvant	103 (4.5)	42 (4.2)	28 (3.6)	33 (6.5)
**Definitive Chemoradiation, *n* = 783**				
Definitive chemoradiation alone	192 (24.5)	6 (46.2)	59 (37.6)	127 (20.7)
Neoadjuvant	14 (1.8)	1 (7.7)	4 (2.5)	9 (1.5)
Adjuvant	543 (69.3)	6 (46.2)	93 (59.2)	444 (72.4)
Neoadjuvant + adjuvant	34 (4.3)	--	1 (0.6)	33 (5.4)
**Definitive Radiation, *n* = 320**				
Definitive radiation alone	153 (47.8)	11 (91.7)	31 (62.0)	111 (43.0)
Neoadjuvant	14 (4.4)	--	2 (4.0)	12 (4.7)
Adjuvant	130 (40.6)	--	15 (30.0)	115 (44.6)
Neoadjuvant + adjuvant	23 (7.2)	1 (8.3)	2 (4.0)	20 (7.8)
**No Definitive Treatment, *n* = 1458**				
No treatment	1336 (91.6)	481 (94.9)	330 (92.5)	552 (93.1)
Neoadjuvant	95 (6.5)	26 (5.2)	28 (7.5)	41 (6.9)

**Table 4:** Neoadjuvant/adjuvant line names by treatment group. Definitive treatment groups defined by color: Primary Surgery, light blue; Definitive Radiation, teal; Definitive Chemoradiation, red; No Definitive Treatment, green.

Primary Surgery				
**Stage at Diagnosis**	**Any Stage**	**Stage IB**	**Stage II**	**Stage IIIA**
Neoadjuvant, *n* = 187				
Clinical Study Drug + ICI/Chemo/Other (*n*, %)	20 (10.7)	7 (8.9)	4 (7.2)	9 (16.1)
ICI + Platinum Doublet (*n*, %)	81 (43.3)	32 (40.5)	33 (60.0)	16 (28.6)
Platinum Doublet (*n*, %)	39 (20.9)	13 (16.5)	8 (14.6)	18 (32.1)
Target (*n*, %)	11 (5.9)	7 (8.9)	3 (5.5)	1 (1.8)
Other (*n*, %)	36 (19.3)	20 (25.3)	4 (7.2)	12 (21.4)
Adjuvant *n* = 1116				
ICI (*n*, %)	59 (5.3)	20 (8.5)	24 (4.7)	15 (4.0)
Platinum Doublet (*n*, %)	843 (75.5)	106 (45.1)	431 (84.5)	306 (82.5)
Target (*n*, %)	81 (7.3)	39 (16.6)	19 (3.7)	23 (6.2)
Other (*n*, %)	133 (11.9)	70 (29.8)	36 (7.1)	27 (7.3)
**Definitive Chemoradiation**				
Neoadjuvant, *n* = 48				
ICI + Platinum Doublet (*n*, %)	8 (16.7)	--	--	8 (19.1)
Platinum Doublet (*n*, %)	28 (58.3)	1 (100.0)	2 (40.0)	25 (59.5)
Other (*n*, %)	12 (25.0)	--	3 (60.0)	9 (21.4)
Adjuvant, *n* = 577				
ICI (*n*, %)	556 (96.4)	6 (100.0)	90 (95.7)	460 (96.4)
Other (*n*, %)	21 (3.6)	--	4 (4.3)	17 (3.6)
**Definitive Radiation**				
Neoadjuvant, *n* = 37				
ICI + Platinum Doublet (*n*, %)	13 (35.1)	--	3 (75.0)	10 (31.3)
Platinum Doublet (*n*, %)	18 (48.6)		--	18 (56.3)
Other (*n*, %)	6 (16.2)	1 (100.0)	1 (25.0)	4 (12.5)
Adjuvant, *n* = 153				
ICI (*n*, %)	132 (86.3)	--	10 (58.8)	122 (90.4)
Other (*n*, %)	21 (13.7)	1 (100.0)	7 (41.2)	13 (9.6)
**No Definitive Treatment**				
Neoadjuvant, *n* = 95				
Chemotherapy (*n*, %)	7 (7.4)	4 (15.4)	2 (7.4)	1 (2.6)
ICI (*n*, %)	6 (6.3)	2 (7.7)	2 (7.4)	2 (5.3)
ICI + Platinum Doublet (*n*, %)	13 (13.7)	1 (3.8)	4 (14.8)	8 (21.1)
Platinum Doublet (*n*, %)	36 (37.9)	--	9 (33.3)	27 (71.1)
Target (*n*, %)	7 (7.4)	2 (7.7)	4 (14.8)	--
Other (*n*, %)	36 (19.3)	17 (65.4)	6 (22.2)	

## Data Availability

The data that support the findings of this study were originated by and are the property of Flatiron Health, Inc., which has restrictions prohibiting the authors from making the dataset publicly available. Requests for data sharing by license or by permission for the specific purpose of replicating results in this manuscript can be submitted to PublicationsDataAccess@flatiron.com.

## Supplementary Materials

The following supporting information can be downloaded at: https://www.mdpi.com/article/10.3390/curroncol32040239/s1, Table S1: Line names in other (under 5% threshold). Table S2: Line name categorization, Table S3: Drugs included in line name categories. Table S4: 1-year Survival by Definitive Treatment Group and Neoadjuvant/Adjuvant Systemic Therapy Use. Table S5: 2-year Survival by Definitive Treatment Group and Neoadjuvant/Adjuvant Systemic Therapy Use. Figure S1. Real World 1 and 2-Year Survival by Definitive Treatment Groups Stratified by Neoadjuvant/Adjuvant Systemic Therapy.

## Abbreviations

The following abbreviations are used in this manuscript:

eNSCLC	Early non-small cell lung cancer
IO	Immunotherapy
ICI	Immune checkpoint inhibitor
TKI	Tyrosine kinase inhibitor
EGFR	Epidermal Growth Factor Receptor
ALK	Anaplastic Lymphoma Kinase
PDL1	Programmed-death Ligand 1
OS	Overall survival
EFS	Event-free survival
EHR	Electronic health record
ICD	International Classification of Diseases
NCCN	National Comprehensive Cancer Network
Plt	Platinum-based chemotherapy
ADJ	Adjuvant
NEO	Neoadjuvant
