# Supplementary material for: Real-World Treatment Patterns and Outcomes Among Patients with Early Non-Small Cell Lung Cancer
Source: Curr Oncol. 2025 Apr 19;32(4):239. doi: 10.3390/curroncol32040239 (PMC12026175; doi:10.3390/curroncol32040239)
Supplement: Supplementary file 1 [file curroncol-32-00239-s001.zip › curroncol-3525056-supplementary.pdf]

SUPPLEMENTAL FIGURES AND TABLES

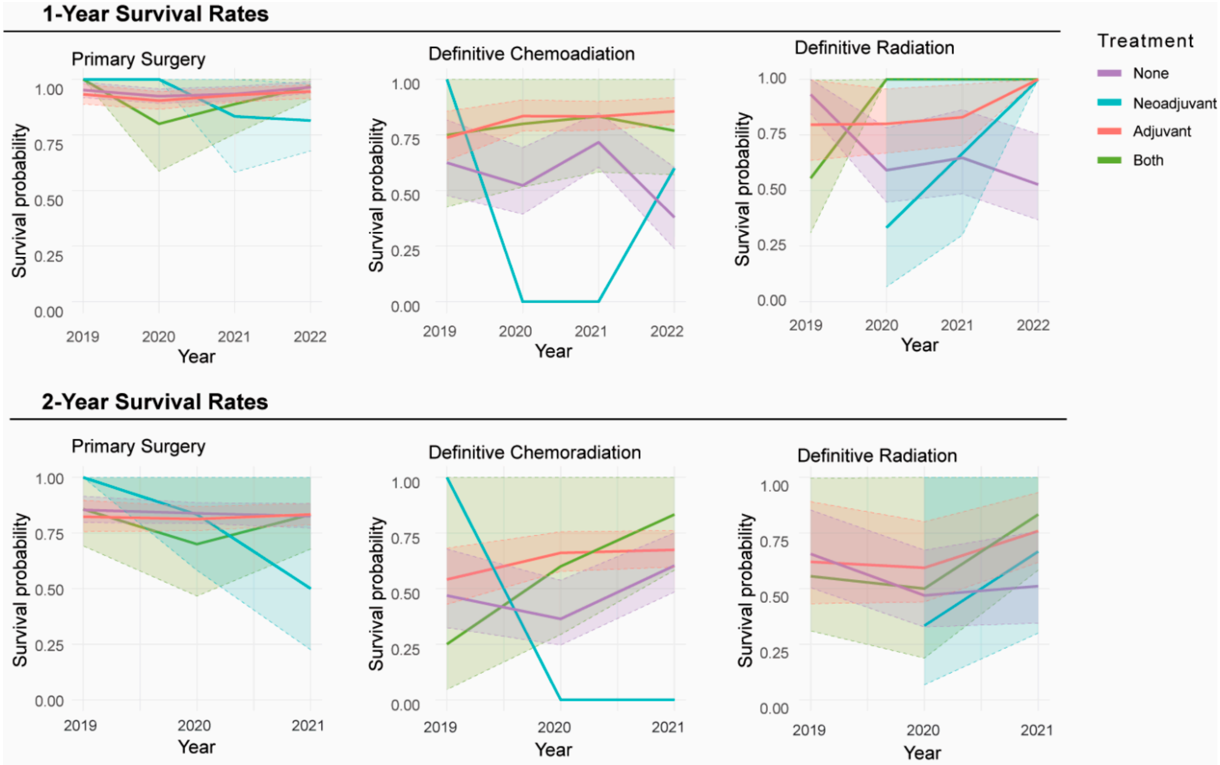

Supplemental Figure S1. Real World 1 and 2-Year Survival by Definitive Treatment Groups Stratified by Neoadjuvant / Adjuvant Systemic Therapy.

Supplemental Table S1: Line Names in Other (under 5% threshold)			
**Definitive Treatment Group**	**Adjuvant / Neoadjuvant**	**Line Name**	**N (%)**
Primary Surgery	Neoadjuvant	Chemotherapy	7 (3.7)
		Clinical Study Drug + Platinum / Platinum Doublet	2 (1.1)
		ICI	2 (1.1)
		ICI + Target	1 (0.5)
		IO	2 (1.1)
		IO + Target	1 (0.5)
		Platinum	2 (1.1)
		Target + Chemo / Other	3 (1.6)
		Target + Platinum Doublet	1 (0.5)
		Other	15 (8.0)
	Adjuvant	Chemotherapy	23 (2.1)
		Clinical Study Drug + ICI / Chemo / Other	13 (1.2)
		Clinical Study Drug + Platinum / Platinum Doublet	5 (0.4)
		ICI + Platinum Doublet	26 (2.3)
		IO	7 (0.6)
		IO + Chemo / Other	2 (0.2)
		IO + Target / Chemo	1 (0.1)

		Other	39 (3.5)
		Platinum	3 (0.3)
		Platinum Doublet / Other	5 (0.4)
		Target + Chemo / Other	5 (0.4)
		Target + Platinum Doublet	4 (0.4)
Definitive Chemoradiation	Neoadjuvant	Chemotherapy	1 (2.1)
		Clinical Study Drug + ICI / Chemo / Other	1 (2.1)
		ICI	1 (2.1)
		Platinum	1 (2.1)
		Target	2 (4.2)
		Target + Chemo / Other	1 (2.1)
		Other	5 (10.4)
	Adjuvant	Clinical Study Drug + ICI / Chemo / Other	3 (0.5)
		ICI	5 (0.9)
		ICI + Chemo / Other	1 (0.2)
		ICI + Platinum	1 (0.2)
		ICI + Platinum Doublet	4 (0.7)
		Other	1 (0.2)
		Platinum Doublet	2 (0.3)
		Target	5 (0.9)
Definitive Radiation	Neoadjuvant	Chemotherapy	1 (2.7)
		IO	1 (2.7)
		IO + Platinum Doublet	1 (2.7)
		Other	1 (2.7)
		Platinum Doublet / Other	1 (2.7)
		Target + Platinum Doublet	1 (2.7)
	Adjuvant	Chemotherapy	2 (1.3)
		ICI + Chemo / Other	2 (1.3)
		ICI + Platinum	1 (0.7)
		ICI + Platinum Doublet	3 (2.0)
		IO	1 (0.7)
		IO + Platinum Doublet	1 (0.7)
		Other	3 (2.0)
		Platinum Doublet	6 (3.9)
		Target	2 (1.3)
No Definitive Treatment	Neoadjuvant	ADC + IO / ICI / Chemo	2 (2.1)
		Clinical Study Drug + ICI / Chemo / Other	3 (3.2)
		IO	1 (1.1)
		IO + Chemo / Other	1 (1.1)
		IO + Target	1 (1.1)
		IO + Target / Chemo	1 (1.1)
		Other	11 (11.6)
		Platinum	3 (3.2)
		Target + Chemo / Other	3 (3.2)

Supplemental Table S2: Line Name Categorization		
**Immune Checkpoint Inhibitors (ICIs)**	**+ None**	• Durvalumab<br>• Pembrolizumab<br>• Atezolizumab<br>• Nivolumab<br>• Cemiplimab<br>• Avelumab<br>• Ipilimumab<br>• Durvalumab,Tremelimumab-Actl<br>• Ipilimumab, Nivolumab
	**+ Platinum**	• Cisplatin,Pembrolizumab

		• Cisplatin,Durvalumab<br>• Carboplatin,Pembrolizumab
	**+ IO**	• Pembrolizumab,Rituximab<br>• Ipilimumab,Nivolumab,Rituximab-Pvvr<br>• Bendamustine,Pembrolizumab,Rituximab-Abbs
	**+ Chemo /Other**	• Cemiplimab,Pemetrexed<br>• Atezolizumab,Leuprolide<br>• Abiraterone,Durvalumab,Leuprolide<br>• Lenalidomide,Nivolumab<br>• Nivolumab,Pemetrexed<br>• Leuprolide,Nivolumab<br>• Pembrolizumab,Pemetrexed<br>• Bicalutamide,Leuprolide,Nivolumab<br>• Docetaxel,Nivolumab<br>• Letrozole,Pembrolizumab<br>• Decitabine,Nivolumab<br>• Nivolumab,Paclitaxel<br>• Pembrolizumab,Vinorelbine<br>• Anastrozole,Paclitaxel Protein-Bound,Pembrolizumab<br>• Gemcitabine,Pembrolizumab<br>• Durvalumab,Pemetrexed<br>• Docetaxel,Pembrolizumab<br>• Durvalumab,Leuprolide<br>• Durvalumab,Tamoxifen<br>• Apalutamide,Durvalumab,Leuprolide<br>• Durvalumab,Letrozole,Tamoxifen<br>• Atezolizumab,Tamoxifen<br>• Leuprolide,Pembrolizumab<br>• Anastrozole,Durvalumab<br>• Fluorouracil,Irinotecan,Leucovorin,Pembrolizumab<br>• Cyclophosphamide,Doxorubicin,Pembrolizumab<br>• Capecitabine,Durvalumab<br>• Durvalumab,Letrozole<br>• Durvalumab,Hydroxyurea<br>• Durvalumab,Paclitaxel<br>• Atezolizumab,Letrozole<br>• Anastrozole,Durvalumab,Gemcitabine,Paclitaxel Protein-Bound<br>• Gemcitabine,Paclitaxel,Pembrolizumab<br>• Paclitaxel Protein-Bound,Pembrolizumab<br>• Paclitaxel,Pembrolizumab<br>• Bicalutamide,Nivolumab<br>• Durvalumab,Gemcitabine,Paclitaxel Protein-Bound<br>• Capecitabine,Durvalumab,Lapatinib<br>• Cemiplimab,Nilotinib,Pemetrexed<br>• Durvalumab,Paclitaxel Protein-Bound,Tremelimumab-Actl<br>• Anastrozole,Pembrolizumab,Trastuzumab-Qyyp
	**+Platinum Doublet**	• Carboplatin,Gemcitabine,Nivolumab<br>• Carboplatin,Pembrolizumab,Pemetrexed<br>• Cisplatin,Nivolumab,Pemetrexed<br>• Carboplatin,Nivolumab,Pemetrexed<br>• Carboplatin,Docetaxel,Nivolumab<br>• Carboplatin,Nivolumab,Paclitaxel<br>• Carboplatin,Paclitaxel,Pembrolizumab<br>• Carboplatin,Nivolumab,Paclitaxel Protein-Bound<br>• Atezolizumab,Carboplatin,Etoposide<br>• Atezolizumab,Cisplatin,Pemetrexed<br>• Atezolizumab,Carboplatin,Pemetrexed<br>• Cisplatin,Gemcitabine,Nivolumab

		• Carboplatin,Durvalumab,Paclitaxel<br>• Cisplatin,Fluorouracil,Pembrolizumab<br>• Atezolizumab,Cisplatin,Etoposide<br>• Cemiplimab,Cisplatin,Pemetrexed<br>• Carboplatin,Docetaxel,Durvalumab<br>• Cisplatin,Pembrolizumab,Pemetrexed<br>• Carboplatin,Durvalumab,Etoposide<br>• Cisplatin,Nivolumab,Paclitaxel<br>• Carboplatin,Durvalumab,Pemetrexed<br>• Atezolizumab,Carboplatin,Paclitaxel<br>• Cisplatin,Paclitaxel,Pembrolizumab<br>• Carboplatin,Paclitaxel Protein-Bound,Pembrolizumab<br>• Fluorouracil,Leucovorin,Oxaliplatin,Pembrolizumab<br>• Cisplatin,Docetaxel,Pembrolizumab<br>• Cisplatin,Docetaxel,Nivolumab,Paclitaxel<br>• Carboplatin,Gemcitabine,Pembrolizumab<br>• Atezolizumab,Carboplatin,Paclitaxel Protein-Bound<br>• Atezolizumab,Carboplatin,Paclitaxel Protein-Bound<br>• Carboplatin,Docetaxel,Pembrolizumab<br>• Carboplatin,Cemiplimab,Paclitaxel<br>• Atezolizumab,Bevacizumab-Awwb,Carboplatin,Paclitaxel<br>• Carboplatin,Nivolumab,Paclitaxel,Pemetrexed<br>• Carboplatin,Paclitaxel,Pembrolizumab,Pemetrexed<br>• Carboplatin,Docetaxel,Paclitaxel,Pembrolizumab<br>• Carboplatin,Leucovorin,Pembrolizumab,Pemetrexed<br>• Carboplatin,Etoposide,Pembrolizumab<br>• Cemiplimab,Cisplatin,Paclitaxel<br>• Capecitabine,Carboplatin,Paclitaxel,Pembrolizumab<br>• Nivolumab,Oxaliplatin,Paclitaxel<br>• Carboplatin,Durvalumab,Paclitaxel,Tremelimumab-Actl<br>• Carboplatin,Cemiplimab,Paclitaxel,Pembrolizumab<br>• Carboplatin,Durvalumab,Pembrolizumab,Pemetrexed<br>• Carboplatin,Ipilimumab,Nivolumab,Pemetrexed<br>• Carboplatin,Nivolumab,Pembrolizumab,Pemetrexed<br>• Carboplatin,Docetaxel,Ipilimumab,Nivolumab<br>• Carboplatin,Gemcitabine,Ipilimumab,Nivolumab<br>• Carboplatin,Durvalumab,Paclitaxel,Pembrolizumab<br>• Carboplatin,Ipilimumab,Nivolumab,Paclitaxel<br>• Atezolizumab,Cisplatin,Durvalumab,Pemetrexed<br>• Atezolizumab,Bevacizumab,Carboplatin,Pembrolizumab,Pemetrexed<br>• Carboplatin,Cemiplimab,Pemetrexed
	**+ Targeted Therapy**	• Axitinib,Pembrolizumab<br>• Atezolizumab,Crizotinib<br>• Atezolizumab,Carfilzomib<br>• Acalabrutinib,Pembrolizumab<br>• Olaparib,Pembrolizumab<br>• Osimertinib,Pembrolizumab<br>• Pembrolizumab,Ribociclib<br>• Ibrutinib,Nivolumab<br>• Lenvatinib,Pembrolizumab<br>• Neratinib,Niraparib,Pembrolizumab<br>• Atezolizumab,Bevacizumab-Awwb<br>• Atezolizumab,Bevacizumab<br>• Pembrolizumab,Ramucirumab<br>• Atezolizumab,Bevacizumab-Bvzr<br>• Bevacizumab-Awwb,Pembrolizumab

	**+ Targeted + Platinum Doublet**	• Atezolizumab,Bevacizumab,Carboplatin,Paclitaxel<br>• Bevacizumab-Bvzr,Fluorouracil,Leucovorin,Oxaliplatin,Trastuzumab-Qyyp<br>• Atezolizumab,Bevacizumab-Awwb,Carboplatin,Paclitaxel Protein-Bound<br>• Atezolizumab,Bevacizumab-Bvzr,Carboplatin,Pemetrexed<br>• Atezolizumab,Bevacizumab-Bvzr,Carboplatin,Paclitaxel<br>• Atezolizumab,Bevacizumab-Adcd,Carboplatin,Paclitaxel Protein-Bound<br>• Bortezomib,Carboplatin,Daratumumab/Hyaluronidase-Fihj,Pembrolizumab,Pemetrexed<br>• Cemiplimab,Cisplatin,Nilotinib,Pemetrexed
**Immunotherapy**	**+ None**	• Rituximab-Pvvr<br>• Rituximab<br>• Rituximab/Hyaluronidase<br>• Rituximab-Abbs<br>• Rituximab-Arrx<br>• Teclistamab-Cqyv<br>• Amivantamab-Vmjw<br>• Daratumumab<br>• Daratumumab/Hyaluronidase-Fihj<br>• Bcg Vaccine<br>• Daratumumab/Hyaluronidase-Fihj,Pomalidomide<br>• Daratumumab,Pomalidomide
	**+ Platinum Doublet**	• Carboplatin,Paclitaxel,Rituximab-Pvvr<br>• Carboplatin,Cetuximab,Paclitaxel<br>• Carboplatin,Etoposide,Ifosfamide,Mesna,Rituximab-Arrx<br>• Carboplatin,Pemetrexed,Rituximab-Pvvr
	**+ Targeted Therapy**	• Bortezomib,Daratumumab/Hyaluronidase-Fihj<br>• Carfilzomib,Daratumumab<br>• Bortezomib,Daratumumab<br>• Ixazomib,Pomalidomide<br>• Carfilzomib,Pomalidomide
	**+ Target + Chemo**	• Carfilzomib,Daratumumab/Hyaluronidase-Fihj,Pomalidomide<br>• Bortezomib,Daratumumab,Lenalidomide<br>• Bortezomib,Daratumumab/Hyaluronidase-Fihj,Lenalidomide<br>• Carfilzomib,Cyclophosphamide,Daratumumab/Hyaluronidase-Fihj<br>• Cyclophosphamide,Pomalidomide<br>• Carfilzomib,Cyclophosphamide,Pomalidomide
	**+ Other / Chemo**	• Daratumumab,Lenalidomide<br>• Elotuzumab,Lenalidomide<br>• Cyclophosphamide,Doxorubicin,Methotrexate,Rituximab-Pvvr,Vincristine<br>• Bendamustine,Rituximab-Arrx<br>• Bendamustine,Rituximab<br>• Bendamustine,Rituximab-Pvvr<br>• Bendamustine,Rituximab/Hyaluronidase<br>• Cyclophosphamide,Doxorubicin,Rituximab-Pvvr,Vincristine<br>• Cyclophosphamide,Doxorubicin,Rituximab-Arrx,Vincristine<br>• Leucovorin,Methotrexate,Rituximab-Pvvr,<br>• Obinutuzumab,Venetoclax,<br>• Cyclophosphamide,Doxorubicin,Rituximab-Abbs,Vincristine,<br>• Daratumumab/Hyaluronidase-Fihj,Lenalidomide<br>• Lenalidomide,Rituximab-Pvvr,Tafasitamab-Cxix
**Targeted Therapy**	**+ None**	• Osimertinib<br>• Ruxolitinib<br>• Dasatinib<br>• Asciminib<br>• Olaparib<br>• Acalabrutinib<br>• Palbociclib<br>• Ibrutinib

		• Vismodegib<br>• Axitinib<br>• Regorafenib<br>• Erdafitinib<br>• Neratinib<br>• Sotorasib<br>• Zanubrutinib<br>• Lenvatinib<br>• Alpelisib<br>• Afatinib<br>• Adagrasib<br>• Nintedanib<br>• Carfilzomib<br>• Pazopanib<br>• Abemaciclib<br>• Umbralisib<br>• Alectinib<br>• Imatinib<br>• Entrectinib<br>• Fedratinib<br>• Bortezomib<br>• Nilotinib<br>• Capmatinib<br>• Lorlatinib<br>• Tepotinib<br>• Sunitinib<br>• Pralsetinib<br>• Erlotinib<br>• Crizotinib<br>• Selpercatinib<br>• Brigatinib<br>• Larotrectinib<br>• Dabrafenib<br>• Ibrutinib,Osimertinib<br>• Dabrafenib,Trametinib<br>• Erlotinib,Osimertinib<br>• Binimetinib,Encorafenib<br>• Osimertinib,Ruxolitinib<br>• Cetuximab<br>• Trastuzumab-Anns<br>• Bevacizumab<br>• Pertuzumab,Trastuzumab-Anns<br>• Pertuzumab,Trastuzumab<br>• Pertuzumab,Trastuzumab-Dkst<br>• Bevacizumab-Awwb<br>• Bevacizumab,Niraparib<br>• Bevacizumab,Niraparib,Olaparib<br>• Bevacizumab-Awwb,Sotorasib<br>• Everolimus<br>• Enzalutamide<br>• Venetoclax<br>• Darolutamide<br>• Fulvestrant<br>• Fulvestrant,Olaparib<br>• Fulvestrant,Palbociclib
	**+ Platinum Doublet**	• Bevacizumab-Awwb,Fluorouracil,Leucovorin,Oxaliplatin<br>• Bevacizumab-Bvzr,Carboplatin,Pemetrexed<br>• Bevacizumab-Awwb,Carboplatin,Pemetrexed

		• Bevacizumab,Carboplatin,Pemetrexed<br>• Bevacizumab-Bvzr,Fluorouracil,Leucovorin,Oxaliplatin<br>• Bevacizumab-Maly,Carboplatin,Paclitaxel<br>• Bevacizumab-Bvzr,Carboplatin,Paclitaxel<br>• Fluorouracil,Leucovorin,Oxaliplatin,Trastuzumab-Dttb<br>• Bevacizumab-Bvzr,Carboplatin,Paclitaxel,Pemetrexed<br>• Bevacizumab-Awwb,Carboplatin,Paclitaxel<br>• Bevacizumab-Bvzr,Capecitabine,Oxaliplatin<br>• Carboplatin,Docetaxel,Pertuzumab,Trastuzumab-Anns<br>• Bevacizumab,Carboplatin,Paclitaxel<br>• Anastrozole,Bevacizumab,Carboplatin,Paclitaxel<br>• Enzalutamide,Fluorouracil,Oxaliplatin<br>• Carboplatin,Enzalutamide,Pemetrexed<br>• Carboplatin,Osimertinib,Pemetrexed<br>• Bortezomib,Carboplatin,Lenalidomide,Paclitaxel<br>• Capecitabine,Carboplatin,Lapatinib,Pemetrexed<br>• Carboplatin,Nintedanib,Pemetrexed<br>• Carboplatin,Osimertinib,Paclitaxel<br>• Carboplatin,Gemcitabine,Ibrutinib
	**+ Other / Chemo**	• Enzalutamide,Leuprolide<br>• Darolutamide,Leuprolide<br>• Enzalutamide,Triptorelin<br>• Abiraterone,Degarelix,Enzalutamide<br>• Enzalutamide,Relugolix<br>• Everolimus,Paclitaxel,Ramucirumab<br>• Anastrozole,Paclitaxel,Trastuzumab-Anns<br>• Bevacizumab-Awwb,Capecitabine<br>• Bevacizumab,Gemcitabine<br>• Docetaxel,Ramucirumab<br>• Capecitabine,Trastuzumab-Qyyp<br>• Bevacizumab-Awwb,Fluorouracil,Leucovorin<br>• Letrozole,Pertuzumab,Trastuzumab-Anns<br>• Paclitaxel,Trastuzumab-Anns<br>• Bevacizumab-Awwb,Temozolomide<br>• Anastrozole,Pertuzumab,Trastuzumab<br>• Exemestane,Pertuzumab,Trastuzumab-Anns<br>• Anastrozole,Letrozole,Pertuzumab,Trastuzumab-Qyyp<br>• Bevacizumab,Pemetrexed<br>• Bevacizumab,Docetaxel,Gemcitabine<br>• Bevacizumab-Awwb,Pemetrexed<br>• Docetaxel,Gemcitabine,Necitumumab<br>• Paclitaxel Protein-Bound,Ramucirumab<br>• Paclitaxel,Ramucirumab<br>• Anastrozole,Bevacizumab,Niraparib<br>• Cetuximab,Fluorouracil<br>• Enzalutamide,Leuprolide<br>• Enzalutamide,Triptorelin<br>• Abiraterone,Degarelix,Enzalutamide<br>• Enzalutamide,Relugolix<br>• Azacitidine,Venetoclax<br>• Bortezomib,Cyclophosphamide,Doxorubicin Pegylated Liposomal<br>• Letrozole,Palbociclib<br>• Carfilzomib,Cyclophosphamide<br>• Bortezomib,Methotrexate<br>• Exemestane,Osimertinib<br>• Bortezomib,Cyclophosphamide<br>• Bortezomib,Lenalidomide<br>• Osimertinib,Tamoxifen

		• Osimertinib,Paclitaxel Protein-Bound<br>• Bicalutamide,Lenvatinib,Leuprolide<br>• Anastrozole,Ibrutinib<br>• Letrozole,Osimertinib<br>• Anastrozole,Osimertinib<br>• Anastrozole,Palbociclib<br>• Fluorouracil,Vismodegib<br>• Bortezomib,Enzalutamide,Leuprolide,Paclitaxel<br>• Abemaciclib,Fulvestrant<br>• Doxorubicin Pegylated Liposomal,Olaparib<br>• Abemaciclib,Anastrozole<br>• Acalabrutinib,Gemcitabine<br>• Leuprolide,Selpercatinib<br>• Gemcitabine,Olaparib<br>• Sotorasib,Tamoxifen
**Platinum**	**+ None**	• Carboplatin<br>• Cisplatin<br>• Oxaliplatin
**Platinum Doublet**	**+ None**	• Carboplatin,Oxaliplatin,Paclitaxel<br>• Capecitabine,Carboplatin,Paclitaxel<br>• Cisplatin,Etoposide<br>• Carboplatin,Docetaxel<br>• Cisplatin,Pemetrexed<br>• Cisplatin,Docetaxel<br>• Carboplatin,Paclitaxel<br>• Cisplatin,Gemcitabine<br>• Carboplatin,Pemetrexed<br>• Cisplatin,Paclitaxel<br>• Carboplatin,Docetaxel,Paclitaxel Protein-Bound<br>• Carboplatin,Paclitaxel Protein-Bound<br>• Carboplatin,Gemcitabine<br>• Carboplatin,Docetaxel,Paclitaxel<br>• Carboplatin,Gemcitabine,Paclitaxel<br>• Carboplatin,Etoposide,Paclitaxel<br>• Carboplatin,Vinorelbine<br>• Cisplatin,Vinorelbine<br>• Carboplatin,Paclitaxel,Pemetrexed<br>• Cisplatin,Docetaxel,Gemcitabine<br>• Carboplatin,Leuprolide,Paclitaxel<br>• Carboplatin,Fluorouracil<br>• Carboplatin,Etoposide<br>• Carboplatin,Gemcitabine,Pemetrexed<br>• Cisplatin,Gemcitabine,Pemetrexed<br>• Cisplatin,Gemcitabine,Methotrexate<br>• Cisplatin,Fluorouracil<br>• Oxaliplatin,Pemetrexed<br>• Cisplatin,Etoposide,Paclitaxel<br>• Capecitabine,Oxaliplatin<br>• Cisplatin,Hydroxyurea,Pemetrexed<br>• Carboplatin,Docetaxel,Gemcitabine<br>• Cisplatin,Paclitaxel Protein-Bound<br>• Carboplatin,Docetaxel,Fluorouracil<br>• Carboplatin,Gemcitabine,Tamoxifen
	**+ Other**	• Apalutamide,Carboplatin,Paclitaxel<br>• Fluorouracil,Leucovorin,Oxaliplatin<br>• Anastrozole,Cisplatin,Pemetrexed<br>• Anastrozole,Carboplatin,Paclitaxel,<br>• Carboplatin,Letrozole,Paclitaxel

		• Fluorouracil,Irinotecan,Leucovorin,Oxaliplatin<br>• Bicalutamide,Cisplatin,Leuprolide,Pemetrexed<br>• Carboplatin,Leucovorin,Pemetrexed<br>• Cisplatin,Leuprolide,Pemetrexed<br>• Carboplatin,Letrozole,Paclitaxel,Tamoxifen<br>• Abiraterone,Cisplatin,Leuprolide,Pemetrexed<br>• Abiraterone,Carboplatin,Paclitaxel<br>• Cisplatin,Pemetrexed,Tamoxifen<br>• Carboplatin,Exemestane,Paclitaxel Protein-Bound<br>• Carboplatin,Letrozole,Paclitaxel Protein-Bound
**Chemotherapy**	**+ None**	• Hydroxyurea<br>• Methotrexate<br>• Topotecan<br>• Fluorouracil<br>• Gemcitabine<br>• Paclitaxel<br>• Capecitabine<br>• Eribulin<br>• Docetaxel<br>• Carmustine<br>• Lurbinectedin<br>• Paclitaxel Protein-Bound<br>• Pemetrexed<br>• Irinotecan<br>• Cyclophosphamide<br>• Romidepsin<br>• Azacitidine<br>• Melphalan<br>• Temozolomide<br>• Vinorelbine<br>• Doxorubicin<br>• Cladribine,Cytarabine<br>• Capecitabine,Gemcitabine<br>• Fluorouracil,Mitomycin<br>• Gemcitabine,Paclitaxel Protein-Bound<br>• Cyclophosphamide,Docetaxel<br>• Docetaxel,Gemcitabine<br>• Gemcitabine,Paclitaxel<br>• Cyclophosphamide,Doxorubicin<br>• Capecitabine,Temozolomide<br>• Capecitabine,Mitomycin<br>• Gemcitabine,Vinorelbine<br>• Gemcitabine,Hydroxyurea<br>• Bleomycin,Dacarbazine,Doxorubicin,Vinblastine<br>• Eflornithine
	**+ Other**	• Docetaxel,Leuprolide<br>• Leucovorin,Methotrexate<br>• Exemestane,Lenalidomide<br>• Lenalidomide,Melphalan<br>• Bicalutamide,Capecitabine,Leuprolide<br>• Anastrozole,Gemcitabine,Paclitaxel Protein-Bound<br>• Cyclophosphamide,Docetaxel,Letrozole<br>• Busulfan,Cyclophosphamide,Fludarabine,Mesna
**Other**<br>**+ None**		• Tamoxifen<br>• Apalutamide

		• Tretinoin<br>• Leuprolide<br>• Exemestane<br>• Bicalutamide<br>• Triptorelin<br>• Porfimer<br>• Leucovorin<br>• Anastrozole<br>• Letrozole<br>• Degarelix<br>• Lenalidomide<br>• Bicalutamide,Leuprolide<br>• Apalutamide,Leuprolide<br>• Abiraterone<br>• Anastrozole,Exemestane<br>• Abiraterone,Leuprolide<br>• Degarelix,Relugolix<br>• Anastrozole,Letrozole<br>• Abiraterone,Bicalutamide,Leuprolide<br>• Exemestane,Letrozole
**Antibody-Drug Conjugate**	**+ None**	• Sacituzumab Govitecan-Hziy<br>• Brentuximab Vedotin<br>• Enfortumab Vedotin-Ejfv<br>• Fam-Trastuzumab Deruxtecan-Nxki<br>• Fam-Trastuzumab Deruxtecan-Nxki,Letrozole
	**+IO / ICI / Chemo**	• Brentuximab Vedotin,Dacarbazine,Doxorubicin,Vinblastine<br>• Cyclophosphamide,Doxorubicin,Polatuzumab Vedotin-Piiq,Rituximab-Pvvr<br>• Enfortumab Vedotin-Ejfv,Pembrolizumab
**Clinical Study Drug**	**+IO / ICI / Chemo**	• Clinical Study Drug,Docetaxel,Pembrolizumab<br>• Clinical Study Drug,Pembrolizumab<br>• Clinical Study Drug<br>• Bicalutamide,Clinical Study Drug,Leuprolide<br>• Clinical Study Drug,Mesna
	**+ Platinum /Platinum Doublet**	• Carboplatin,Clinical Study Drug<br>• Cisplatin,Clinical Study Drug,Pemetrexed<br>• Carboplatin,Clinical Study Drug,Pemetrexed<br>• Carboplatin,Clinical Study Drug,Paclitaxel

Supplemental Table S3: Drugs Included in Line Name Categories	
Chemotherapy	Azacitidine, Bendamustine, Bleomycin, Busulfan, Capecitabine, Carmustine, Cladribine, Cyclophosphamide, Cytarabine, Dacarbazine, Decitabine, Docetaxel Doxorubicin, Doxorubicin Pegylated Liposomal, Eflornithine, Eribulin, Etoposide, Fludarabine, Fluorouracil, Gemcitabine, Hydroxyurea, Ifosfamide, Irinotecan, Lurbinectedin, Melphalan, Methotrexate, Mitomycin, Paclitaxel, Pemetrexed, Temozolomide, Topotecan, Vinblastine, Vinorelbine
Platinum Chemotherapy	Carboplatin, Cisplatin, Oxaliplatin
Immunotherapy	Amivantamab-Vmjw, Bcg Vaccine, Daratumumab, Daratumumab/Hyaluronidase-Fihj, Elotuzumab, Obinutuzumab, Rituximab, Tafasitamab-Cxix, Teclistamab-Cqyv, Pomalidomide
Targeted	Abemaciclib, Acalabrutinib, Adagrasib, Afatinib, Alectinib, Alpelisib, Asciminib, Axitinib, Bevacizumab, Binimetinib, Bortezomib, Brigatinib Capmatinib Carfilzomib Cetuximab Crizotinib, Dabrafenib, Dasatinib, Darolutimide, Encorafenib, Entrectinib, Enzalutamide, Erdafitinib, Erlotinib, Everolimus, Fedratinib, Fulvestrant, Ibrutinib, Imatinib, Lapatinib, Larotrectinib, Lenvatinib, Lorlatinib, Necitumumab, Neratinib, Nilotinib, Nintedanib, Niraparib, Olaparib, Osimertinib, Palbociclib, Pazopanib, Pertuzumab, Pralsetinib, Ramucirumab, Regorafenib, Ribociclib, Romidepsin, Ruxolitinib, Selpercatinib, Sotorasib, Sunitinib, Tepotinib, Trametinib, Trastuzumab-Qyyp, Umbralisib, Venetoclax, Vismodegib, Zanubrutinib
Immune Checkpoint inhibitors	Atezolizumab, Avelumab, Cemiplimab, Durvalumab, Ipilimumab, Nivolumab, Pembrolizumab, Tremelimumab-Actl
ADCs	Brentuximab Vedotin, Enfortumab Vedotin-Ejfv, Fam-Trastuzumab Deruxtecan-Nxki, Polatuzumab Vedotin-Piiq, Sacituzumab Govitecan-Hziy
Other	Anastrozole, Apalutamide, Bicalutamide, Degarelix, Exemestane, Lenalidomide, Letrozole, Leucovorin, Leuprolide, Mesna, Abiraterone, Porfimer, Relugolix, Tamoxifen, Tretinoin, Triptorelin

Supplemental Table S4: 1-year Survival by Definitive Treatment Group and Neoadjuvant / Adjuvant Systemic Therapy Use				
	2019	2020	2021	2022
Surgery alone	95.2% (91.8 - 98.7%), n = 156	92.4% (89.2 – 95.8%), n = 275	93.4% (90.1 – 96.8%), n = 246	96.5% (93.9 - 99.1%), n = 236
+ Neoadjuvant	100.0% (100.0 - 100.0%), n = 10	100.0% (100.0 - 100.0%), n = 9	83.3% (58.2 - 100.0%), n = 7	81.5% (67.8 – 98.9%), n = 34
+ Adjuvant	93.3% (88.9 – 97.9%), n = 124	90.4% (86.5 – 94.5%), n = 217	92.9% (89.7 – 96.2%), n = 254	94.5% (91.6 - 97.6%), n = 228
+ Neoadjuvant and Adjuvant	100.0% (100.0 - 100.0%), n = 14	80.0% (58.7 - 100.0%), n = 10	88.9% (75.5 - 100.0%), n = 18	96.9% (91.0 - 100.0%), n = 35
Definitive Chemoradiation alone	62.5% (47.8%, 81.7%), n = 32	52.3% (39.4%, 69.3%), n = 44	71.7% (60.5%, 84.9%), n = 53	37.9% (23.8%, 60.4%), n = 29
+ Neoadjuvant	100.0% (100.0%, 100.0%), n = 1	--	--	60.0% (29.3%, 100.0%), n = 5
+ Adjuvant	73.8% (63.5%, 85.7%), n = 61	83.5% (76.8%, 90.8%), n = 109	83.3% (77.1%, 90.1%), n = 126	85.6% (79.8%, 91.8%), n = 132
+ Neoadjuvant and Adjuvant	75.0% (42.6%, 100.0%), n = 4	80.0% (51.6%, 100.0%), n = 5	83.3% (58.3%, 100.0%), n = 6	76.9% (57.1%, 100.0%), n = 13
Definitive Radiation alone	93.2% (84.6 – 100.0%, n = 32	59.1% (44.7 – 78.2%), n = 37	64.7% (48.5 – 86.3%), n = 29	52.3% (36.8– 75.5%), n = 33
+ Neoadjuvant	--	33.3% (6.7- 100.0%), n = 3	66.7% (30.0 – 100.0%), n = 3	100.0% (100.0 – 100.0%), n = 3
+ Adjuvant	79.6% (63.6 – 100.0%), n = 21	80.0% (66.9 – 95.7%), n = 30	83.0% (70.4 – 97.8%), n = 30	100.0% (100.0 – 100.0%), n = 28
+ Neoadjuvant and Adjuvant	55.6% (31.0 – 99.7%), n = 9	100.0% (100.0 – 100.0%), n = 4	100.0% (100.0 – 100.0%), n = 6	100.0% (100.0 – 100.0%), n = 3

Supplemental Table S5: 2-year Survival by Definitive Treatment Group and Neoadjuvant / Adjuvant Systemic Therapy Use			
	2019	2020	2021
Surgery alone	85.4% (79.6 – 91.5%), n = 156	83.9% (79.4 – 88.7%), n = 275	82.6% (77.3 – 88.2%), n = 246
+ Neoadjuvant	100.0% (100.0 - 100.0%), n = 10	83.3% (58.3 - 100.0%), n = 9	50.0% (22.5 - 100.0%), n = 7
+ Adjuvant	82.3% (75.6 – 89.7%), n = 124	81.3% (76.1 – 86.8%), n = 217	83.4% (78.7 - 88.4%), n = 254
+ Neoadjuvant and Adjuvant	85.7% (69.2 - 100.0%), n = 14	70.0% (46.7 – 100.0%), n = 10	83.3% (67.8 - 100.0%), n = 18
Definitive Chemoradiation alone	46.9% (32.4%, 67.8%), n = 32	36.4% (24.6%, 53.8%), n = 44	60.3% (48.5%, 75.1%), n = 53
+ Neoadjuvant	100.0% (100.0%, 100.0%), n = 1	--	--
+ Adjuvant	54.1% (42.9%, 68.2%), n = 61	66.1% (57.7%, 75.6%), n = 109	67.4% (59.7%, 76.1%), n = 126
+ Neoadjuvant and Adjuvant	25.0% (4.6%, 100.0%), n = 4	60.0% (29.3%, 100.0%), n = 5	83.3% (58.3%, 100.0%), n = 6
Definitive Radiation alone	65.6% (50.4 – 85.4%), n = 32	46.9% (32.7 – 67.3%), n = 37	51.1% (34.5 - 75.8%), n = 29
+ Neoadjuvant	--	33.3% (6.7 - 100.0%), n = 3	66.7% (30.0 – 100%), n = 3
+ Adjuvant	62.0% (43.2 – 89.1%), n = 21	59.4% (44.0 – 80.1%), n = 30	75.9% (61.8 – 93.2%), n = 30
+ Neoadjuvant and Adjuvant	55.6% (31.0 - 99.7%), n = 9	50.0% (18.8 - 100.0%), n = 4	83.3% (58.3 - 100.0%), n = 6
